# Posterior eyespots in larval chitons have a molecular identity similar to anterior cerebral eyes in other bilaterians

**DOI:** 10.1186/s13227-015-0036-0

**Published:** 2015-12-22

**Authors:** Oliver Vöcking, Ioannis Kourtesis, Harald Hausen

**Affiliations:** Sars International Centre for Marine Molecular Biology, University of Bergen, Thormøhlensgate 55, 5008 Bergen, Norway; Department of Biology, University of Bergen, Thormøhlensgate 55, 5008 Bergen, Norway

**Keywords:** Eyes, Evolution, Development, Replication, Mollusks

## Abstract

**Background:**

Development of cerebral eyes is generally based on fine-tuned networks and closely intertwined with the formation of brain and head. Consistently and best studied in insects and vertebrates, many signaling pathways relaying the activity of eye developmental factors to positional information in the head region are characterized. Though known from several organisms, photoreceptors developing outside the head region are much less studied and the course of their development, relation to cerebral eyes and evolutionary origin is in most cases unknown. To explore how position influences development of otherwise similar photoreceptors, we analyzed the molecular characteristics of photoreceptors we discovered at the very anterior, the posttrochal mid-body and posterior body region of larval *Leptochiton asellus*, a representative of the chiton subgroup of mollusks.

**Results:**

Irrespective of their position, all found photoreceptors exhibit a molecular signature highly similar to cerebral eye photoreceptors of related animals. All photoreceptors employ the same subtype of visual pigments (r-opsin), and the same key elements for phototransduction such as *GNAq*, *trpC* and *arrestin* and intracellular r-opsin transport such as *rip11* and *myosinV* as described from other protostome cerebral eyes. Several transcription factors commonly involved in cerebral eye and brain development such as *six1/2*, *eya, dachshund, lhx2/9* and *prox* are also expressed by all found photoreceptor cells, only *pax6* being restricted to the anterior most cells. Coexpression of *pax6* and *MITF* in photoreceptor-associated shielding pigment cells present at the mid-body position matches the common situation in cerebral eye retinal pigment epithelium specification and differentiation. Notably, all photoreceptors, even the posterior ones, further express clear anterior markers such as *foxq2, irx, otx*, and *six3/6* (only the latter absent in the most posterior photoreceptors), which play important roles in the early patterning of the anterior neurogenic area throughout the animal kingdom.

**Conclusions:**

Our data suggest that anterior eyes with brain-associated development can indeed be subject to heterotopic replication to developmentally distinct and even posterior body regions. Retention of the transcriptional activity of a broad set of eye developmental factors and common anterior markers suggests a mode of eye development induction, which is largely independent of body regionalization.

**Electronic supplementary material:**

The online version of this article (doi:10.1186/s13227-015-0036-0) contains supplementary material, which is available to authorized users.

## Background

Eyes are one of the best studied animal sensory organs. Despite the great variety of photoreceptive structures, from simple pigment cup eyes to lens eyes of vertebrates or complex eyes of arthropods, the employment of numerous orthologues transcription factors in development is striking. This led to the idea of a common origin of many kinds of eyes [1–4]. Nonetheless, the evolutionary significance of molecular components employed in the formation of non-ubiquitous eye components is still controversially debated [5–8] as well as the possible composition and organization of ancestral eyes [9, 10].

In bilaterian animals, the discussion strongly focuses on cerebral eyes. Eye development is here embedded within the developmentally distinct neurogenic area of the head region and eye specification and differentiation is well known to generally depend on both, multilayered and self-regulating interactions of cell intrinsic transcription factors, but also regional intracellular signaling and induction [11–13]. Little attention so far has been paid to photoreceptors occurring in other body regions though they are known from several animal groups [14]. One reason may be that they often are difficult to detect, since they are not necessarily associated with shielding pigmentation. Most analyses focus on morphology, while molecular data of non-cephalic photoreceptors are scarce and the question of possible homology to cerebral eyes is unsolved.

In most cases it is an open question, whether the main factors driving specification and terminal differentiation of cerebral eyes are also active in non-cephalic photoreceptors. Thus, we carried out for the first time a broad molecular characterization of an eye developing outside the neurogenic head region and chose a representative of the mollusk subgroup of chitons (Polyplacophora) as study object. As in many animal groups, the eyes of these organisms form already in the larval phase, but they occur in an unusual position. In trochophore larvae of related taxa like gastropods or annelids, the eyes form anterior to the first ciliary ring, the prototroch, which in this kind of larva marks the hind end of the neurogenic prospective head region [15–18], while the eyes in chiton larva arise behind the prototroch [19, 20]. Fate maps of embryos with highly stereotyped spiral cleavage further point out the peculiar development of chiton eyes, since these derive from the second [21] and not the first micromere quartet as do the cerebral eyes in gastropods, annelids, nemerteans, or flatworms [17, 18, 22–25]. Both, due to the divergent position and cell lineage homology to eyes of related organisms has been questioned and chiton eye development was suggested to rely on deviant inductive effects [21] caused by a different cellular surrounding of the eye precursor cells.

While screening expression of common eye developmental factors by the chiton eyes, we found also hitherto unknown, extraocular photoreceptors at the very anterior and the posterior end of the chiton larva. Surprisingly, all photoreceptors share irrespective of their position in the embryo a nearly identical molecular inventory similar to that of cerebral eye photoreceptors of other animals having impact on general concepts of eye development and evolution.

## Methods

### *Leptochiton asellus* culture

Adult *L.asellus* were collected close to the coastline of Bergen, Norway, during September–December 2011–2014. Adult animals were cultured at 8 °C covered from light in groups of males and females and fertilized egg balls were collected each morning. Larvae were kept at 18 °C on a 12 h/12 h light/dark cycle.

### RNA-Seq and transcriptome assembly

Total RNA was extracted from cryofixed 2–11-day-old larvae using the Agencourt RNAdvance Tissue Kit (Beckman Coulter). Library preparation and sequencing was performed by EMBL Genomics Core Facility (Heidelberg, Germany) using cation-based chemical fragmentation of RNA, Illumina Truseq RNA-Sample Preparation Kit and 1 lane of 100 bp paired end read sequencing on Illumina HiSeq 2000. Raw reads were trimmed and error corrected with Cutadapt 1.2.1 [26], the ErrorCorrectReads tool implemented in Allpaths-LG [27] and assembled with Trinity [28].

### Gene cloning and RNA probe preparation

Contig sequences for the investigated genes were identified in the transcriptome data set by bidirectional blast. Whole transcripts or fragments were amplified by PCR with specific primers from cDNA prepared with SuperScript III (Invitrogen), ligated into pgemT-easy vector (Promega) and cloned into Top10 chemically competent *E. coli* (Invitrogen). Clone sequences were verified by Sanger sequencing and the *Las*-*r*-*opsin* sequence was elongated by Rapid amplification of cDNA ends with the SMARTer RACE cDNA Amplification KIT (Clontech). DIG- and FITC-labeled sense and antisense RNA probes were generated from plasmid DNA with Megascript Kit (Ambion) or with T7- and SP6-RNA Polymerases (Roche).

### Gene orthology and phylogenetic analyses

Reciprocal blast yielded unambiguous results for gene orthology assignment of *Las*-*eya*, *Las*-*dachshund, Las*-*arrestin, Las*-*otx*, *Las*-*t23d, Las*-*nk2.1* and *Las*-*frizzled5/8*. In all other cases, public databases (Genebank, JGI, Uniprot) and the *Leptochiton* transcriptome were screened for homologs by text search, blast and HMMER with respective query sequences or domain profiles for subsequent gene tree generation based on maximum likelihood and bayesian inference (see supplementary information for details). Accession numbers: *Las*-*r*-*opsin* (KU193716), *Las*-*arrestin* (KU193717*), Las*-*rip11* (KU193718), *Las*-*foxq2 A* (KU193719), *Las*-*foxq2 B* (KU193720), *Las*-*GNAq* (KU193721), *Las*-*irx A* (KU193722), *Las*-*irx B* (KU193723), *Las*-*klf* (KU193724), *Las*-*lhx2/9* (KU193725), *Las*-*MITF* (KU193726), *Las*-*myosinV* (KU193727), *Las*-*ovo* (KU193728), *Las*-*pax6* (KU193729), *Las*-*prox* (KU193730), *Las*-*six1/2* (KU193731), *Las*-*six3/6 A* (KU193732), *Las*-*six3/6 B* (KU193733), *Las*-*sp6/9* (KU193734), *Las*-*trpC* (KU193735), *Las*-*tyrosinase A* (KU193736), *Las*-*tyrosinase B* (KU193737), *Las*-*frizzled5/8* (KU193738), *Las*-*nk2.1* (KU193739), *Las*-*t23d* (KU193740), *Las*-*dachshund* (KU193741), *Las*-*eya* (KU193742), *Las*-*otx* (KU193743).

### Immunohistochemistry

Custom-made polyclonal antibodies against the peptides ARPISVMRKMGHKRA and VKAVADHEKEMHNMAKRL from the cytosolic loops between the transmembrane domains III and IV and V and VI of *Las*-*r*-*opsin* were raised in guinea pig and affinity purified by 21st Century Biochemicals (Marlboro, USA). To assure antigen specificity, both peptide sequences were blasted against the *Leptochiton* transcriptome and gave the single existent r-opsin as the only hit. Only the antibody affinity purified with the peptide VKAVADHEKEMHNMAKRL gave clear signals and was used for the experiments. Antibody specificity was tested by dot blots and preadsorption negative controls (see Additional file 1 and Additional file 2: Figure SI for further details). Stainings were performed according to the protocol of [29] with some adjustments. Animals were fixed for 2 h in 4 % PFA in buffer PBS (0.05 M PB/0.3 M NaCl/0.1 % Tween; pH 7.4) at room temperature. After fixation animals were washed and stored at 4 °C in PBS before being transferred to THT (0.1 M Tris, 0.1 % Tween 20). The custom-made polyclonal guinea pig anti-Las-r-opsin primary antibody was applied for 48–72 h 1:100 diluted in THT containing 5 % sheep serum at 4 °C. Then, specimens were washed (2 × 10 min) in 1 M NaCl in THT and in THT (5 × 30 min), before being incubated with Alexa Fluor 488 goat anti-guinea pig IgG (Sigma, Saint Louis, USA) diluted 1:500 in THT at 4 °C for 24 h. Specimens were stored in embedding medium (90 % glycerol/1× PBS/0.25 % DABCO) until usage.

### Characterization of shielding pigments

Larvae that already developed eyespots were transferred to different chemicals which dissolve different shielding pigments. To dissolve ommochromes, animals were transferred to acidified methanol and left overnight at 4 °C. The same procedure was done with 0.1 M NaOH to check for pheomelanins and hydrogen peroxide for digesting insoluble eumelanins.

### In situ hybridization

Animals were fixed for 2.5 h in 4 % PFA in phosphate buffer with Tween (PTW; pH 7.4) and stored at −20 °C in Methanol until usage. The in situ hybridization procedure was performed as described by [30] with some modifications (see SI for extended description). In brief, Proteinase K concentration was reduced to 5 ng/ml, hybridization buffer contained 5 % dextran sulfate, the incubation time was 72 h and stainings were done with a combination of Fast Blue (Sigma-Aldrich) and Fast Red (Roche) as described for Zebrafish [31]. To evaluate staining, significance control experiments with sense probes were made. Additionally, we combined in situ hybridization and antibody stainings, by processing specimen after in situ hybridization with the immunohistochemistry procedure aforementioned.

### Light microscopy

Light microscopic images were taken using a Nikon Eclipse E800 and a Nikon AZ100M microscope and adjusted with Photoshop CS5. Confocal images were taken with a Leica SP5 confocal microscope and the image stacks processed with ImageJ and Photoshop CS5.

### Electron microscopy

Sample preparation was done according to the protocol given in [32] with some modifications. In brief, larvae were fixed in 2.5 % glutaraldehyde in sodium cacodylate buffer, postfixed in 1 % Osmium tetroxide, en-bloc stained with reduced Osmium, dehydrated in a graded ethanol series and embedded in Epon/Araldite (see Additional file 1 for further details). Serial sections of 70 nm were cut with an ultra 35° diamond knife (Diatome, Biel, Switzerland) on an ultramicrotome (Leica EM UC7) and collected on Synaptek Beryllium-Copper slot grids (Electron Microscopy Sciences) coated with Pioloform (Ted Pella) and counterstained with 2 % uranyl acetate and lead citrate. Complete series were imaged with STEM-in-SEM similar as described by [33] at a resolution of 8 nm/pixel in a ZEISS Supra 55VP equipped with ZEISS Atlas for automated large field of view imaging (see Additional file 3 for details). Acquired images were processed with Adobe Photoshop CS5, first registered rigidly followed by affine and elastic alignment [34] with TrakEM2 [35] implemented in Fiji. 3D reconstructions were performed by assigning area lists for the nuclei and cell surface and balls for pigment granules. Final 3D modeling and rendering was done with Blender.

## Results

### The same type of opsin employed in most protostome cerebral eyes is expressed in the posttrochal eyes, but also in anterior and posterior extraocular photoreceptors of larval *Leptochiton asellus*

**Fig. 1:**
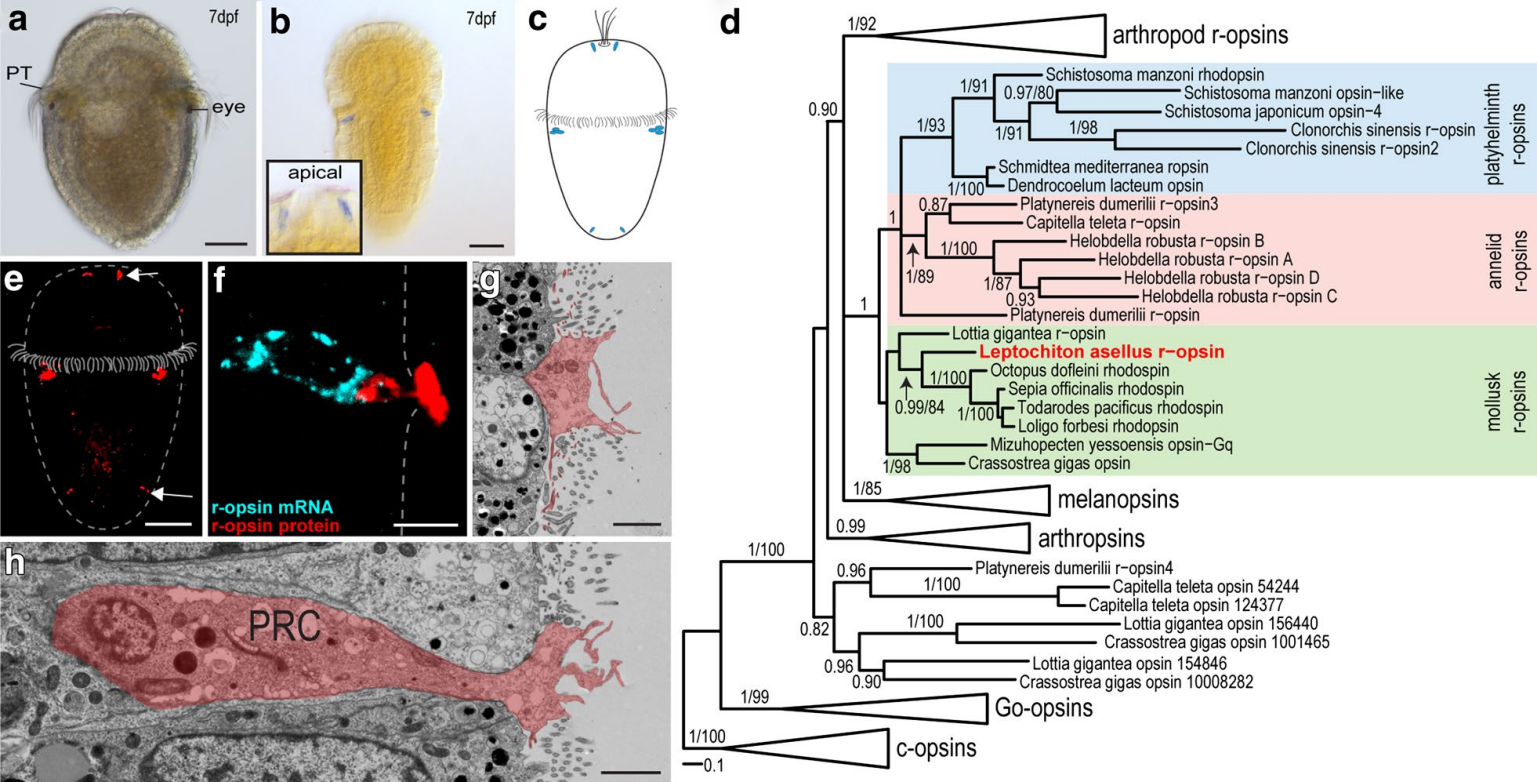
R-opsin expression in 7 dpf *L. asellus* larvae and eye photoreceptor cell morphology. **a** Larva with eyespot posterior to the prototroch (PT). **b** Expression of *Las*-*r*-*opsin* in the eye and the apical area (*inlet*). **c** Scheme of *Las*-*r*-*opsin* expression. **d** *Las*-*r*-*opsin* groups well supported within mollusk and other protostome r-opsins. Support values are given as Bayesian posterior probability/maximum likelihood bootstrap values (see “Methods” and Additional file 3 for uncollapsed tree and details of tree inference). **e** Specifically designed *Las*-*r*-*opsin* antibody reveals r-opsin protein in the eyes, as well as in apical and posterior cells (*arrows*). **f** Combination of *Las*-*r*-*opsin* in situ hybridization (*cyan*) and antibody staining (*red*) showing the spatial separation of the *Las*-*r opsin* protein in the apical area (*right side*) and the mRNA expression in the nucleus of an eye sensory cell. **g**, **h** Electron microscopic images of the photoreceptor cells (PRC) showing the flask-shaped morphology and the apical microvillar extensions. Apical surface on the *right*. (*Scalebars* 100 μm in **a**, **b**, **e**; 5 μm in **f**; 2 μm in **g**, **h**. PRC photoreceptor cell)

Opsins play a fundamental role in light transduction in cerebral eye photoreceptors of basically all bilaterians. Screening our larval RNA-seq data of *Leptochiton asellus* for r-opsins, the typical opsin type employed in protostome cerebral eyes, we found one clear ortholog grouping together with other mollusk r-opsins in phylogenetic analyses (Fig. 1d, Additional file 4: Figure S2F). Surprisingly, RNA in situ hybridization (ISH) revealed, that this *r*-*opsin* it is not only expressed in the posttrochal eyes, but also in one or more pairs of cells at the anterior end, as well as in cells at the posterior end of the larvae (Fig. 1a–c, Additional file 4: Figure S2E). The anterior *r*-*opsin* + cells and the *r*-*opsin* + cells in the eyes appear in larvae 48–72 h post fertilization (hpf) (Additional file 4: Figure S2A–C), whereas the posterior *r*-*opsin* + cells can only be found from 7 days post fertilization (dpf) onwards and due to small signal size only be detected by confocal microscopy (Additional file 4: Figure S2E). All *r*-*opsin* + cells endure metamorphosis and can still be detected in juvenile animals via ISH (Additional file 4: Figure S2D). The *r*-*opsin* expression in the eye region of the juveniles may explain the light sensitivity of the recently characterized Schwabe organ in adult *L. asellus*, assumed to be the retained larval eye [36, 37]. The ISH results have been confirmed using a specifically designed antibody against the *L. asellusr*-*opsin*, demonstrating that both *r*-*opsin* protein and mRNA are present in all found photoreceptor cells (Fig. 1e). To examine the relation amongst these dispersed *r*-*opsin* + cells, as well as the relation to photoreceptor cells (PRCs) in cerebral eyes of other animals, we undertook a detailed molecular and structural analysis.

### All photoreceptor cells are equipped with elements of the r-opsin typic signaling cascade

**Fig. 2:**
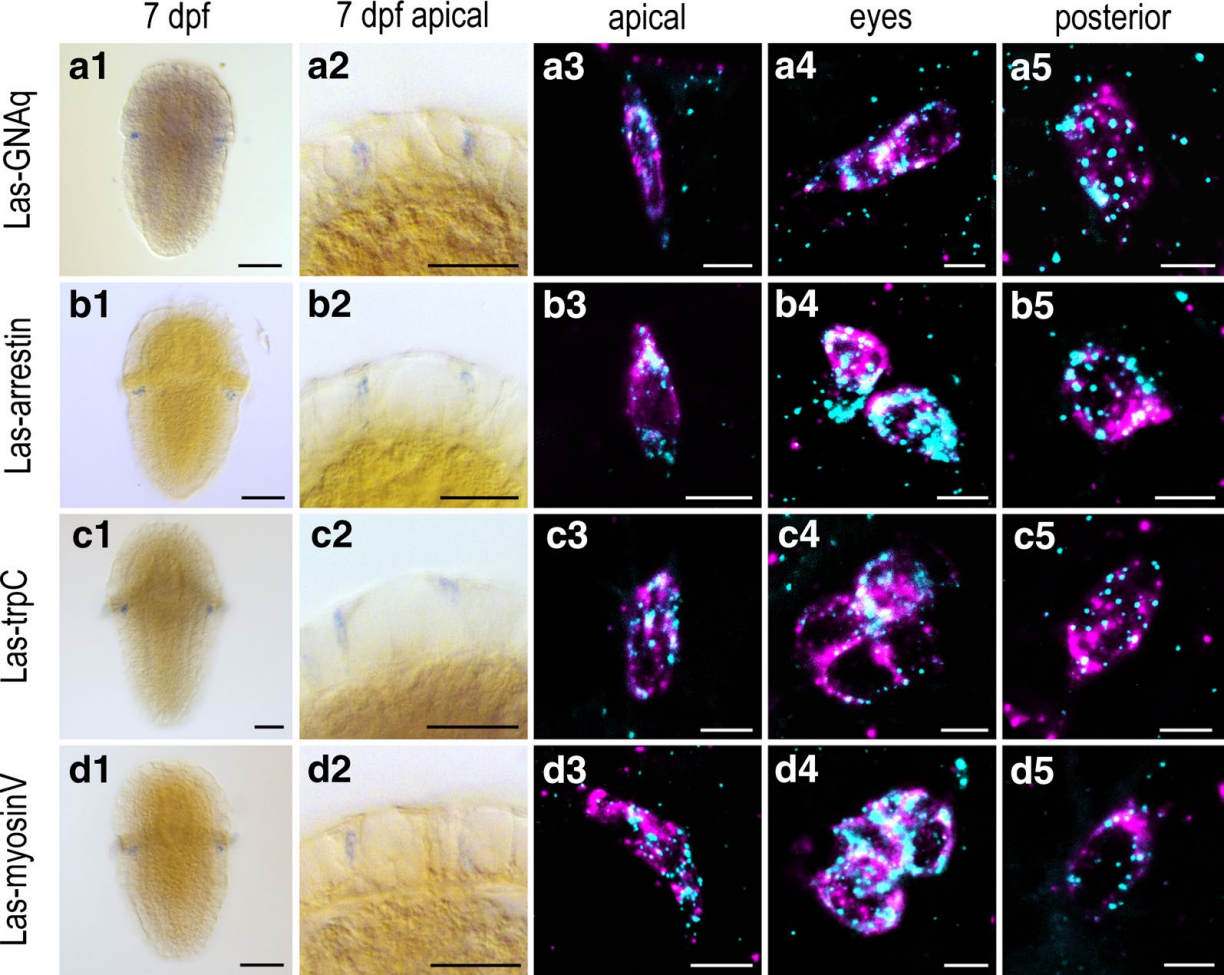
Expression of selected genes involved in phototransduction and opsin transport in 7dpf larva. *Column 1, 2*: single labeling of gene X. *Column 3–5*: double labeling of gene X (*cyan*) and *Las*-*r*-*opsin* (magenta) in the anterior, posttrochal eye and posterior region. *Las*-*GNAq* (**a1**–**a5**), *Las*-*arrestin* (**b1**–b**5**), *Las*-*trpC* (**c1**–**c5**) and *Las*-*myosinV* (**d1**–**d5**) are coexpressed with *Las*-*r*-*opsin* in anterior, eye and posterior PRCs. (*Scalebars*: 100 μm in *column 1*; 50 μm in *column 2*; 5 μm in *columns 3–5*)

R-opsins are G-protein coupled receptors acting via the IP3 signaling pathway. The whole cascade is particularly well studied in *Drosophila* eye PRCs [38] and has been confirmed in many other animals [39, 40]. By means of double in situ hybridization, we found orthologs of several r-opsin typic phototransduction elements expressed in all *r*-*opsin* + cells, i.e., the Gq-Protein α-subunit *GNAq*, a *trpC* channel ortholog and the opsin deactivating *arrestin* (Fig. 2a–c). These data give evidence that all *r*-*opsin* + cells in *L. asellus* are potentially capable of initiating a GNAq-mediated phototransduction cascade, regardless their position in the animal.

### The r-opsin protein is localized in the microvilli bearing apical tip of the PRCs and the cells express r-opsin typical transport elements

By far the most r-opsin-dependent PRCs known show a microvillar (rhabdomeric) organization [41], where the light detecting opsin is incorporated in the membrane of apical sensory microvilli. Serial section STEM imaging of the eye region revealed that the axon bearing sensory cells of the eye send thin dendritic processes to the epidermal surface (Fig. 1g, h). The dendritic tips are expanded, surmount the surrounding cells and bear characteristic cytoplasmic extensions and numerous microvilli traveling horizontally underneath the cuticle. Combining ISH and immunohistochemistry, we could see that the *r*-*opsin* mRNA is localized in the cell body of the PRCs, whereas the *r*-*opsin* protein is restricted to the apical most part of the cells (Fig. 1f) matching well the microvilli bearing tips of the axon bearing eye PRCs seen in the electron microscope (Fig. 1g, h). It is known from Drosophila that active transport of r-opsin loaded vesicles into the microvilli of PRCs is crucial for photoreceptor function and development [42]. We found orthologs of two of the involved transporter molecules, *myosinV* and *rab interacting protein(rip11)*, likewise expressed in all PRCs (Fig. 2d, Additional file 5: Figure S3C) suggesting similar transport mechanisms.

### All PRCs express well-known cerebral eye developmental factors

**Fig. 3:**
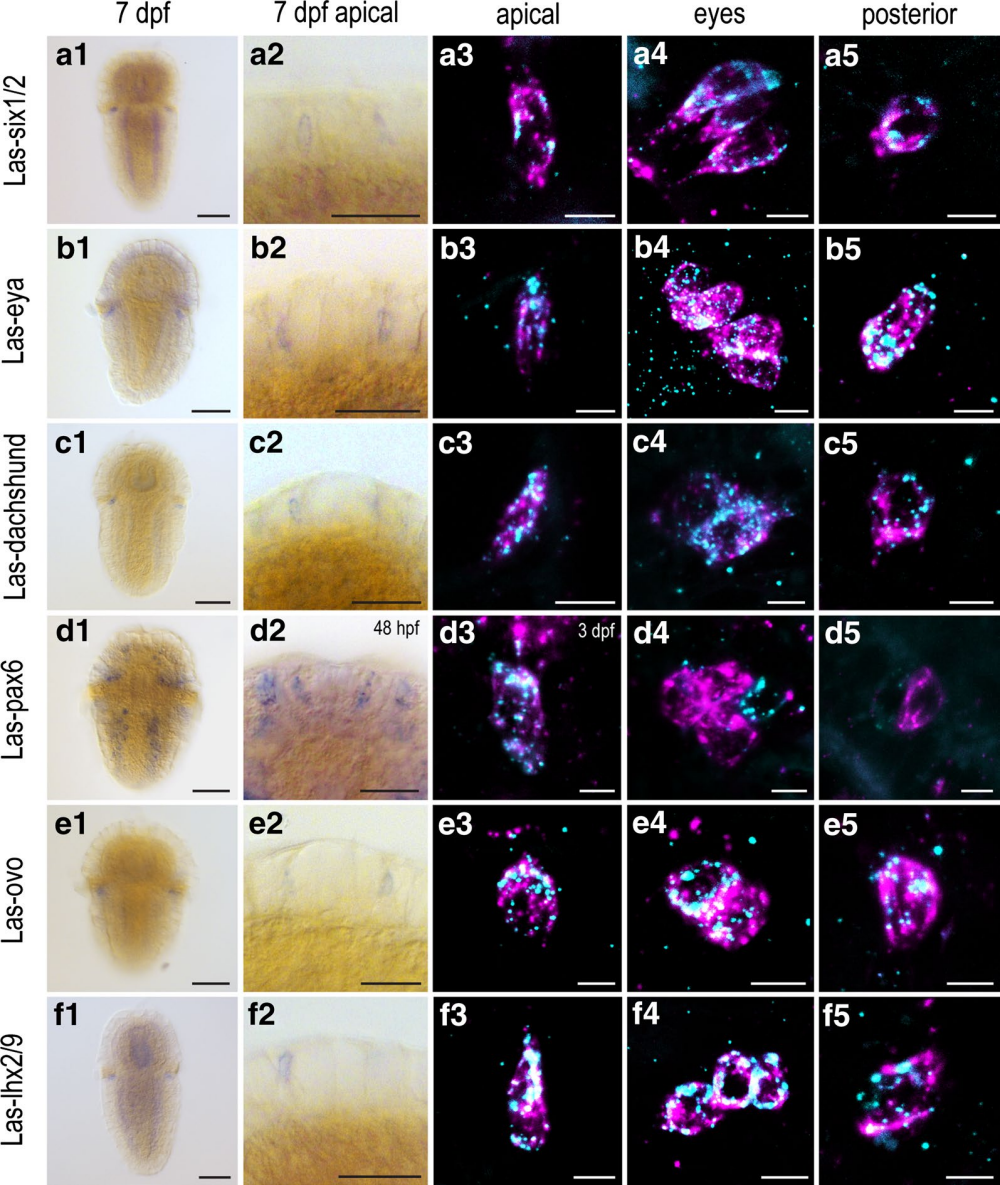
Expression of selected genes involved in photoreceptor cell development in 7dpf larva. *Column 1, 2* single labeling of gene X. *Column 3–5* double labeling of gene X (cyan) and *Las*-*r*-*opsin* (magenta) in the anterior, posttrochal eye and posterior region. **a1**–**a5** Expression of *Las*-*six1/2* in all PRCs as well as in the longitudinal nerve cords (**a1**). **b1**–**b5** Expression of *Las*-*eya* in all PRCs. **c1**–**c5** Expression of *Las*-*dachshund* in all PRCs, as well as a faint expression in the apical area (**c1**). **d1**–**d5** *Las*-*pax6* is broadly expressed in the nervous system (**d1**), otherwise limited to the apical PRCs of young larvae (24–48 hpf) (**d2**, **d3**), not coexpressed with the *Las*-*r*-*opsin* in the eye PRCs, but in adjacent, more distal cell bodies (**d4**), no expression in the posterior PRCs (**d5**). **e1**–**e5** Expression of *Las*-*ovo* in all PRCs. **f1**–**f5** Expression of *Las*-*lhx2/9* in all PRCS, as well as a broader expression in the entire nervous system (**f1**). (*Scalebars* 100 μm in *column 1*; 50 μm in *column 2*; 5 μm in *columns 3*–*5*)

During the last two decades, molecular studies not only suggest a likely common ancestry of bilaterian cerebral eye PRCs but also explored a set of eye developmental genes, employed in a great variety of different animals [1, 5, 43]. Particularly, the combined expression of the widespread eye developmental transcription factors (TFs) *six1/2*, *eya* and *dachshund* in chiton PRCs (Fig. 3a–c) indicate a relation to cerebral eye PRCs of other animals. Also *ovo*, which is crucial for planarian eye development [44], is expressed by the chiton PRCs (Fig. 3e). Surprisingly, expression of all these genes is not restricted to the eye PRCs, but also present in the anterior and posterior PRCs. *Pax6* on the other hand is only expressed in the anterior PRCs during the early developmental stages (24–72 h) (Fig. 3d). It is not coexpressed with *r*-*opsin* in the eye, but expressed in adjacent cells distal to the PRC bodies. No *pax6* expression was detected in the posterior PRCs. Thus, direct involvement of *pax6* in PRC specification seems only likely for the apical cells. To further explore how similar the anterior, posttrochal eye and posterior PRCs are on the level of TF expression, we included in the survey *prox* and *lhx2/9*, known to be involved in a variety of animals in cerebral eye PRC development [45–47] and *klf* critical to maintain the PRC functional [48, 49]. All of them show clear coexpression with *r*-*opsin* in all PRCs (Fig. 3f, Additional file 5: S3A, B) supporting the view that regulatory processes are highly similar in all these cells.

### The shielding pigment cells of the posttrochal eyes show a conserved signature

**Fig. 4:**
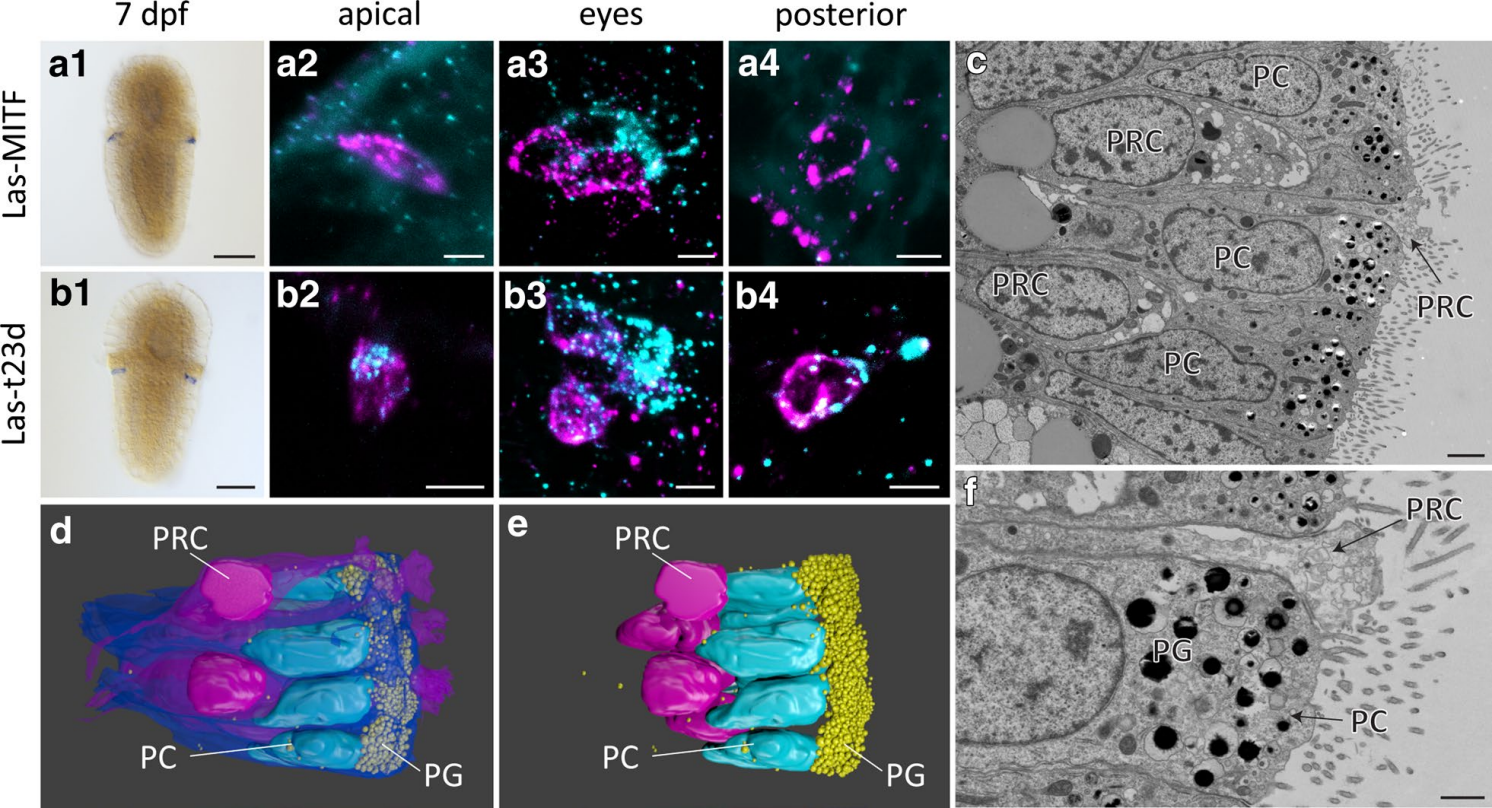
Pigment cell characterization of the eye in 7dpf larva. Expression analysis of *Las*-*MITF* (**a**) and *Las*-*t23d* (**b**) and electron microscopy (**c**–**f**). **a1** *Las*-*MITF* is strongly expressed in the eye region. **a2**–**a4** *Las*-*MITF* (*cyan*) is expressed in cell bodies directly distal (*right side*) to the *Las*-*r*-*opsin* signal (*magenta*) in the eye PRCs, but not in apical, or posterior region. **b1** *Las*-*t23d* is strongly expressed in the eye region. **b2**–**b4** *Las*-*t23d* (*cyan*) is expressed in cell bodies directly distal (*right side*) to the eye PRC *Las*-*r*-*opsin* signal (*magenta*), as well as a faintly expressed in the anterior, eye and posterior PRCs. **c**, **f** The nuclei of the eye PRCs (PRC) are located proximal of the shielding pigment cell nuclei (PC). **d**, **e** 3D reconstruction of the eye, showing the arrangement of pigment and photoreceptor cells, as well as the layer of shielding pigment granules (PG), covering the nuclei of the pigment cells and shielding the apical extensions of the PRCs. (*Scalebars* 100 μm in **a1**, **b1**, 5 μm in **a2**–**a4**, **b2**–**b4**, 2 μm **c** and 1 μm in **f**)

Besides photoreceptors, another essential part of an eye is the shielding pigmentation, which enables directional detection of light by partly covering the photoreceptors. The pigment granula may reside in the PRC itself [50] or are more often located in associated pigment cells [43]. To characterize the pigment cells of the larval eye, we first reconstructed the structure in 3D from our ssSTEM data set. Beside the axon bearing PRCs, we found in fact only one other cell type in the eye, epithelial pigment cells harboring high numbers of electron-dense granules in their apical region (Fig. 4c, f). These granules effectively shield the r-opsin + dendritic tips of the PRCs extending above the pigment cells (Fig. 4d, e). The perikarya of the PRCs, however, take in a basal position and the uppermost layer of cell nuclei in the eye region are those of the pigment cells (Fig. 4d, e), which therefore can be unambiguously identified by its relative position.

The main component of the pigment cell granules seems to be ommochrome, known from eye pigment cells in other molluscs and arthropods [5]. This is due to strong expression of *tryptophan*-*2,3*-*dioxygenase* (*t23d*) involved in ommochrome synthesis [51] distal to the *r*-*opsin* + eye PRC somata (Fig. 4b1, b3) and solubility of the eye pigment spots in acidified methanol as it is characteristic for ommochrome, but not after treatment with sodium hydroxide or hydrogen peroxide dissolving and digesting melanins (Additional file 6: Figure S4) [8]. Interestingly, in some specimen, *t23d* was also weakly expressed in eye PRCs, as well as in anterior and posterior PRCs (Fig. 4b2, b4). Additionally, we observed in all PRCs expression of two paralogs of *tyrosinase* (Additional file 5: Figure S3E, F), known to be involved in melanin synthesis [52] and *sp6/9* (Additional file 5: Figure S3D), which plays a crucial role in planarian eye pigment cup regeneration [53]. Though, general pigment synthesis rate in PRCs is probably low, since we observed only few pigment granules in the PRCs by electron microscopic analysis and we could not observe any kind of pigmentation in PRCs by light microscopy fading by the above-mentioned treatments.

The observed expression sites of *pax6* distally to the larval eye photoreceptors (Fig. 3d4) likewise have to be assigned to transcriptional activity within the eye pigment cells, which is in accordance with the known role of *pax6* in cerebral eye pigment cell development. Further, we found *MITF* expressed in the pigment cells (Fig. 4a), another well-known factor in pigment cell development and pigment synthesis, which has been described to directly interact with *pax6* in pigment cell specification and differentiation [54, 55].

### All PRCs express typical anterior markers such as *foxq*2, *otx* and *irx*

Several TFs involved in cerebral eye development are important patterning genes of the embryonic early anterior territory and show spatially restricted expression. *Foxq2*, *six3/6*, *nk2.1*, *frizzled5/8* and *otx,* for instance, are considered to be strong apical markers, since they show clear expression in the apical area only during early development in different animals [15, 56].

**Fig. 5:**
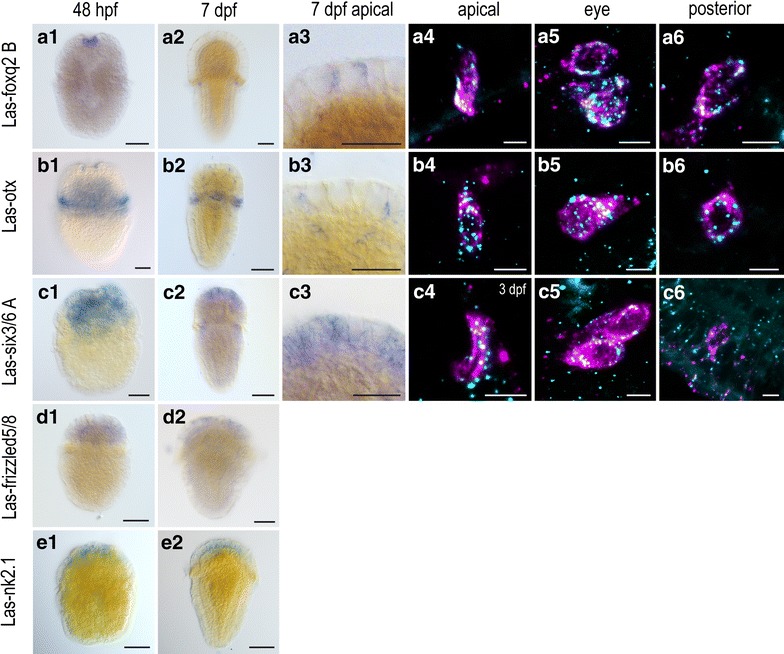
Expression of selected apical markers during the development of *L.asellus*. *Column 1–3* single labeling of gene X. *Column 4–6* double labeling of gene X (cyan) and Las-r-opsin (magenta) in the anterior, posttrochal eye and posterior region. *Las*-*foxq2 B* expression is clearly limited to the apical area in young larvae of 48 hpf (**a1**) and can be found in later developmental stages in all PRCs (**a2**–**a6**). *Las*-*otx* expression in young larvae of 48 h hpf is limited to the apical region and the prototroch (**b1**) and can be found in all PRCs in older developmental stages (**b4**–**b6**). **c1**–**c6** *Las*-*six3/6 A* is expressed in the apical area only in young larvae (48 hpf) (**c1**) but can be found to be expressed in the apical PRCs and some individuals showed faint expression in the eye PRCs (**c5**) of older larvae. No expression was found in the posterior PRCs (**c6**). **d1**–**d3** Expression of *Las*-*frizzled5/8* is limited to the apical area (**d1**). **f1**–**f3** *Las*-*nk2.1* expression is limited to the apical area (*Scalebars* 100 μm in *columns 1, 2*; 50 μm in *column 3*; 5 μm in *columns 4–6*)

Due to the overall similarity of all *L. asellus* PRCs, we thus were interested to see, whether the PRCs differ in the expression of such anterior factors. First, we noted that several genes show a similar anterior expression described in larvae and embryos of other organisms [15, 56, 57]. In early, 2–3-day-old larvae expression of genes such as *nk2.1*, two paralogs of *foxq2*, *otx*, and *frizzled5/8* is clearly restricted to the apical region anterior of the prototroch (with exception of otx, which is as usual also expressed by the trochoblasts) (Fig. 5a, d, e, Additional file 7: Figure S5A). Two paralogs of *six3/6* were detected, one in early stages with broad, the other with scattered expression in the apical area (Fig. 5c, Additional file 7: Figure S5D). In later stages, notably we found both paralogs of *foxq2*, *otx* and two paralogs of *irx*, also expressed not only in the anterior PRCs, but also in the posttrochal eye and the posterior PRCs (Fig. 5a, b, Additional file 7: Figure S5A, B, C). Also faint expression of one *six3/6* paralog in the eyes could be detected in some specimens, though not in the posterior PRCs (Fig. 5c). The other *six3/6* ortholog shows expression in distinct epidermal cells posterior to the eye not expressing *r*-*opsin* (Additional file 7: Figure S5D).

## Discussion

Development of complex traits such as eyes is based upon fine-tuned networks and interactions with the surrounding tissue. In general, formation of animal cerebral eyes is intimately intertwined with the early patterning of the brain region and several mechanisms relaying the activity of cell intrinsic eye developmental factors to positional external signaling are well characterized in systems such as insects and vertebrates [11, 12, 58]. Our data demonstrate the existence of photoreceptors, which are highly similar to cerebral eye PRCs of related animals, located in very different body regions of larvae and juveniles of the chiton *L. asellus*, i.e., as extraocular PRCs at the very anterior and the very posterior end, and forming part of eyes in the posttrochal mid-body region. We conclude that during evolution brain-associated cerebral eyes were replicated in distant areas without obvious changes in their molecular identity and retaining the same transcriptional activity of factors commonly employed in the development of brain-associated cerebral eyes. This addresses important general aspects of eye development and evolution.

### Dispersed occurrence of cerebral eye PRC homologs

First indication for the relation of the investigated PRCs to the predominant type of PRCs present in cerebral eyes of related animals is provided by the visual pigment employed. Coexistence of several ancient opsin types with different signaling cascades and electrophysiological responses has meanwhile been reported in many bilaterian organisms [59–62] and this diversity has probably been an important factor during emergence of several kinds of PRCs enrolled in various functions from simple phototaxis to vision, circadian and lunar rhythmics, light-triggered reproduction, and many other physiological responses.

Out of this variety, the investigated chiton PRCs express a clear ortholog of that opsin type employed in the microvillar PRC type of protostome cerebral eye PRCs, namely *r*-*opsin*. The localization of the *r*-*opsin* protein in the apical, microvilli bearing tips of the cells, the expression of *GNAq, trpC, arrestin, myosinV* and *rip11* indicates the same mode of phototransduction and opsin transport as it is known from the eye PRCs of arthropods, annelids, or flatworms [3, 38, 42, 44, 53, 63–66]. All investigated PRCs express a series of developmental factors such as *six1*/*2, eya, dachshund, otx, prox, lhx2/9, irx and ovo*, which play important roles in the development of cerebral eyes throughout bilaterian animals [1, 3, 5, 44–46]. This multifaceted correspondence between the chiton PRCs and cerebral eye PRCs of other protostomes points towards common evolutionary origin.

### Non-cephalic photoreceptors in Bilateria

Though structural or physiological data suggest existence of non-cephalic photoreceptors in several bilaterians [14, 67, 68], molecular data exist only from few organisms and focus mainly on detection and transduction of the light stimulus. Interrelationships of different non-cephalic PRCs amongst each other and relation to cerebral eye PRCs are thus largely unknown. Due to the variety of existing kinds of visual pigment, many different types of PRCs may be involved in non-cephalic light detection. Indeed, in *C. elegans* and in *Drosophila*, even recruitment of novel light-sensing kinds of G-protein coupled receptors not directly related to opsins has been reported from dermal light sensors [69, 70]. Though, r-opsin has been detected meanwhile in few non-cephalic PRCs of adults, but clarification of their evolutionary origin will need detailed characterization of their development and molecular features. Recently, a possible ancient presence of r-opsin + cells in the bilaterian trunk nervous system has been proposed due to corresponding findings in the ventral nerve cord of the annelid *Platynereis dumerilii* [71] and in the neural tube of *Branchiostoma* (forming part of the Hesse eye cups) [72, 73]. The respective PRCs deviate from cerebral eye PRCs by a likely dependence on *pax2/5/8*. They are regarded as an ancient own type of PRCs with a regulatory developmental network, which is related, but distinct from that of cerebral eye PRCs [71]. In contrast, our data demonstrate a high similarity regarding the expression of developmental genes between all observed PRCs of *L. asellus* and cerebral eye PRCs of other taxa. Relation of all observed PRCs to cerebral eye PRCs is further indicated by the expression of clear anterior markers by all cells (see below for detailed discussion). The virtually molecular identity amongst the *L. asellus* PRCs further indicates that the dispersal was a rather recent evolutionary process not equivalent with the emergence of the above-mentioned bilaterian trunk nervous system PRCs. It may be subject of future work to find out, whether the observed posterior chiton PRCs are more widely distributed across other taxa. To our knowledge, posterior r-opsin + PRCs have not yet been discovered in any bilaterian larvae with the exception of cephalochordate Hesse eye cups, which form already in the larval. In adults, possible candidates are non-cephalic r-opsin + PRCs described from cephalopods such as skin chromatophores [74] or the light-sensitive elements of the bioluminescent organs, though the latter are assumed to have emerged independently within different squid subtaxa [75, 76]. Likewise, the segmental r-opsin + PRCs found in the parapodia of the annelid *Platynereis dumerilii* [71] may share a common origin. However, this remains speculative and needs further comparative investigations.

### Heterotopic replication under retention of the original site-specific characteristics is the most likely scenario

To our knowledge, no similar situation showing such a high correspondence of photoreceptors in different body regions as we observed in *L. asellus* has so far been reported from any other animal. Generally, the anterior, mid and posterior body regions are developmentally distinct areas in eumetazoan animals and this seemingly holds true for the larva of chitons. The observed anterior restriction of the early expression of *six3/6, frizzled 5/8, nk2.1, foxq2* and *otx* (*otx* also in the trochoblasts) in early larvae of *L. asellus* matches the situation in other bilaterians [15, 56, 57, 77–80] and corroborates the general view that the prototroch in trochophore larvae marks the posterior boundary of the anterior neurogenic region [15–18]. Further, the anterior, posttrochal and posterior lateral epidermis harboring the investigated PRCs have a different embryological origin in the spirally cleaving chitons evidenced by cell lineage data in the close relative *Chaetopleura apiculata*. The respective parts of the epidermis arise here from the first, second and third micromere quartet, respectively [21].

An intriguing question is how the distributed occurrence of the very same kind of PRCs was achieved during evolution. One possibility is that the cells arose from a common or close analogue and reached their distinct positions by unanticipated far-distance cell migration. This seems unlikely, since none of the investigated markers traces cell ontogeny back to a common origin. Further, not only the surrounding posttrochal epidermis, but also the eye itself is proposed to have a different clonal origin than the pretrochal and the posterior lateral epidermis in *Chaetopleura apiculata* [21].

Instead, our dataset strongly supports a scenario where the posttrochal and posterior PRCs are heterotopic replicates from original anterior cerebral eye PRCs. Noteworthy, we found with onset of opsin expression in addition to the aforementioned common eye developmental factors two paralogs of *irx* and *foxq2,* as well as one copy of *six3/6* and *otx* likewise present in all investigated PRCs of *L. asellus*, even in the posterior ones (with exception of six3/6 missing in the posterior PRCs). Genes which all are involved in eye development in other organisms [5, 44, 81–83], but which are due to their role in the general patterning of the anterior neuroectoderm usually clearly restricted to the anterior pole of the embryo in response to posterior Wnt signaling [15, 56, 80]. Interestingly, we found *frizzled 5/8*, known to mediate Wnt-dependent downregulation of anterior neuroectoderm factors [79], broadly expressed in the early pretrochal area as it is known from echinoderms, hemichordates, cephalochordates and annelids [15, 80, 84–86], but not in any PRCs, which might explain unresponsiveness to this kind of suppression by the chiton PRCs. Irrespective of the underlying regulation, expression of clear anterior markers strongly suggests inheritance of an ancestral anterior identity by the posttrochal and posterior PRCs.

Two hypotheses are conceivable to explain the distribution of shielding pigments associated to PRCs. First, the capability to synthesize ommochrome, the main shielding component in the *L. asellus* eyes in cells neighboring the posttrochal PRCs was acquired by co-opting the relevant modules for pigment synthesis from other pigmented cells. This cannot be ruled out, since ommochrome is not exclusively restricted to eyes in protostomes such as insects or cephalopods. Noteworthy, however, we could not detect any other spot expressing *t23d* than the eye shielding pigment cells in all investigated stages. As an alternative not only PRCs, but at least once a complete eye was replicated (i.e., PRCs and associated shielding pigment cells). This hypothesis is more likely due to the expression of both, *pax6* and the common pigment cell specification factor *MITF* [1, 5, 87] by the eye shielding pigment cells of *L. asellus,* which hints on descent from an eye pigment cell. Close interaction of both factors is known from *Drosophila* [87] and is recently shown to drive not only specification, but also terminal differentiation of the vertebrate retinal pigment epithelium [55]. To explain the distribution of PRCs and shielding pigment cells in larval *L. asellus*, we suggest the emergence of the posttrochal eye by descent of an ancestral anterior whole eye and secondary loss of eye pigment cells in the anterior region. The anterior PRCs are likely direct remnants of ancestral anterior cerebral eyes as known from relatives as, e.g., gastropods, cephalopods, annelids or flatworms. Both, primary emergence of whole eyes or only PRCs in the posterior region is conceivable.

The potential to form eyes is in spiralians usually restricted to descendants of the first micromere quartet. Due to the unusual clonal origin of the chiton larval eye, segregation of this ability from the first to the second micromere quartet has been assumed in the chiton *Chaetopleura apiculata* [21]. According to our data, the anterior, the posttrochal and the posterior PRCs of *L. asellus* lie in a position comparable to the distribution of micromere 1a/c, respectively, 2a/c and 3a/c descents in *C. apiculata*. Our data thus suggest that the potential to form eyes in *L. asellus* rather has been kept as it is usual in spiral cleaving embryos in the first micromere quartet, but has been extended to the second and probably third micromere quartet.

### General implications for eye development

Replication of eyes under strong retention of the molecular characteristics addresses interesting general aspects of eye and PRC development. Best studied in insects and vertebrates, eye developmental factors are known to interact in a complex self-regulatory network [81, 88]. When experimentally misexpressed, several network components are capable to evoke activity of the whole network and even to induce the development of ectopic eyes [1]. This underlies, however, clear restrictions. In *Drosophila*, successful induction is only possible to subpopulations of imaginal disc cells, which exhibit extreme plasticity and are generally amenable to trans-determination [89, 90]. In vertebrates, overexpression of single factors evokes ectopic eye tissue only nearby the regular eyes and overexpression of a whole cocktail of eye developmental factors is needed to induce eye development in other areas [91]. Consistently, the knowledge about how cell–cell signaling in these organisms relays the activity of cell intrinsic eye developmental factors to positional information and narrowing down the area capable to form eyes and specify retinal cell types is steadily increasing [11, 12, 58, 92]. Comparative investigations in other animal groups will certainly be fruitful to deepen the general understanding of selector gene regulation. Our data suggest that systems like chitons with replicated, highly similar cerebral eye derivatives are interesting subjects to explore how conserved patterns of eye selector gene activity are initiated and regulated in new surroundings and how established positional restrictions are overridden by evolution.

## Conclusions

We discovered photoreceptors, which are distributed across very different body regions of larvae and juveniles of the chiton *L. asellus*, i.e., as extraocular PRCs at the very anterior and the very posterior end, and forming part of eyes in the posttrochal mid-body region. All photoreceptors are highly similar to each other and share numerous molecular characteristics with cerebral eye photoreceptors of other animals regarding phototransduction, subcellular localization and intracellular transport of the visual pigment and the expression of transcription factors commonly involved in photoreceptor specification and differentiation. The shielding pigment cells of the mid-body eyes likewise share molecular characteristics with cerebral eye pigment cells of other organisms. We suggest that the observed photoreceptors arose by heterotopic replication from ancestral cerebral eyes under retention of transcriptional activity of a broad set of eye developmental factors and anterior markers commonly involved in the early patterning of the anterior neurogenic region even in the very posterior photoreceptors. In this kind of embryos with spirally, largely determinant cleavage pattern, this implies extension of the potential to form this kind of photoreceptors from the first to the second and probably also third micromere quartet. While in insect and vertebrate models, many mechanisms are characterized relaying the transcriptional activity of eye selector genes to positional information in the brain region, our data suggest in chitons a mode of eye development induction, which is largely independent of body regionalization. We propose that further studies on systems like chitons with replicates of cerebral eye derivatives may yield interesting insights on how site-specific restrictions of selector gene activity can be overridden by evolution. As outlined by [93] and [94], repeated replication and later divergence may be a more common phenomenon in eye evolution than anticipated and causes complex evolutionary histories similar to the case of gene family evolution. Putting a stronger research focus on non-cephalic photoreceptors will thus be essential for gaining a deeper understanding of the evolution of light sensation.

## Additional files

10.1186/s13227-015-0036-0 Supplementary Methods.
10.1186/s13227-015-0036-0 R-opsin antibody preadsorbtion test. (A1-A3) The negative control of the specifically designed *r*-*opsin* antibody shows no specific signal in the eye region under prototroch (PT) after the specimen and antibody were preadsorbed with the antigenic peptide. **(B1-3)** The positive control of the antibody shows a clear signal in the eye region (Scalebars: 20 µm in A; 15 µm in B).
10.1186/s13227-015-0036-0 Phylogenetic analyses of studied genes with uncertain orthology after reciprocal blast.
10.1186/s13227-015-0036-0 Expression of *Las*-*r*-*opsin* in different developmental stages and r-opsin evolution. **(A)** No expression was found in larvae between 24-48 hpf and first expression was detected in larvae of 3 dpf **(B)**. **(C)** Clear expression of *Las*-*r*-*opsin* in larvae of 4 dpf and in juvenile animals **(D)**, which also show a background staining in the developing shell. **(E)** Expression in posterior photoreceptor cells can first be detected in larvae of 7 dpf and only by confocal imaging of fluorescent signal due to low signal strength. **(F)** Uncollapsed tree of r-opsin analysis shown in Fig. 1. (Scalebars: 100 μm in A-E).
10.1186/s13227-015-0036-0 Expression of genes involved in PRC development, opsin transport and pigment cell markers. Column 1-2: single labelling of gene X. Column 3-5: double labelling of gene X (cyan) and *Las*-*r*-*opsin* (magenta) in the anterior, posttrochal eye and posterior region. **(A1-5)** Expression of *Las*-*klf* in all PRCs **(A1)**. **(B1-5)** Expression of *Las*-*prox* in all PRCs, as well as in the longitudinal nerve cords **(B1)**. **(C1-5)** Expression of *Rab 11 interacting protein* (*Las*-*rip11*) in all PRCs. **(D1, 3-5)** Expression of *Las*-*sp6/9* in all PRCs. **(E** **+** **F 1-5)** Expression of *Las*-*tyrosinase A* + *B* in all PRCs. (Scalebars: 100 μm in column 1; 50 μm in column 2; 5 μm in columns 3-5).
10.1186/s13227-015-0036-0 Shielding pigment test. **(A)** After treating the specimen with acidified Methanol the eye pigment spot completely fades. **(B)** After transferring the animal to 0.1 M NaOH the animal starts disintegrating but the shielding pigment (SP) does not fade and is still clearly visible under the prototroch (PT). The image was taken immediately before the entire animal disintegrated (PT). **(C)** After transferring the animal to a solution of 10 % Hydrogenperoxide the specimen starts to clear up and disintegrate whereas the shielding pigment remains. The image was taken immediately before the entire animal disintegrated (Scalebares 100 µm).
10.1186/s13227-015-0036-0 Expression of apical markers during the development of *L.asellus*. Column 1-3: single labelling of gene X. Column 4-6: double labelling of gene X (cyan) and *Las*-*r*-*opsin* (magenta) in the anterior, posttrochal eye and posterior region. **(A1-6)** Expression of *Las*-*foxq2 A* is limited to the apical area only in young larvae **(A1)** and can be found in all PRCs in older developmental stages. **(B** **+** **C 1-6)** Expression of *Las*-*irx A* and *B* in all PRCs. **(E1-6)** *Las*-*six3/6 B* is expressed in the apical area of young larvae **(E1)** and a clear expression can be found separate from the apical area underneath the eye region in older larvae **(E2, E 5)**. No expression was found in the apical **(E4)** or posterior **(E6)** r-opsin + cells. (Scalebars: 100 μm in columns 1,2; 50 μm in column 3; 5 μm in columns 4-6).

## Abbreviations

ISH: in situ hybridization
PRC: photoreceptor cell
TF: transcription factor
