# Supplementary material for: Posterior eyespots in larval chitons have a molecular identity similar to anterior cerebral eyes in other bilaterians
Source: EvoDevo. 2015 Dec 22;6:40. doi: 10.1186/s13227-015-0036-0 (PMC4689004; doi:10.1186/s13227-015-0036-0)
Supplement: Supplementary file 1 — 10.1186/s13227-015-0036-0 Supplementary Methods. [file 13227_2015_36_MOESM1_ESM.docx]

**Appendix S5 Supplementary Methods**

**Phylogenetic analyses**

Sequences alignments with MAFFT 7.221 (EINSI option) were manually curated. Best fitting substitution models were selected with ProtTest 3.4 according to Akaike information, Bayesian information and Decision theory criteria or with the RAXML based ProteinModelSelection script by Alexandros Stamatakis. Maximum likelihood analyses were run with RAXML 8.0.32 with gamma model of rate heterogeneity and rapid bootstrapping. Bayesian analyses were run with Phylobayes 3.3 using the same substitution models chosen for ML analyses and gamma distribution for rate heterogeneity. Four chains were run and tested for convergence with bcomp command discarding the 20% first cycles as burn-in and used for generating majority-rule consensus trees. Data-set specific GTR substitution matrices have been shown to fit data best for opsin evolution inference [57, 59]. Due to the limited size of our own r-opsin sequence dataset we used the broad opsin sequence data-set of [59] for calculating a custom substitution matrix with RAXML. Best fit to our own data-set was verified with ProtTest by replacing the FLU substitution matrix with the calculated matrix and with Phylobayes by 10 times cross validation against implemented substitution matrices with gamma model of rate heterogeneity.

**Tests for antibody specificity**

**Dot Blot**

To check if the r-opsin antibody binds to the peptide VKAVADHEKEMHNMAKRL (21^st^ Century Biochemicals, Marlboro, USA) we performed a dot blot analysis. Nitrocellulose membrane (pore size 0.2 µm; Biorad) was cut in small pieces and soaked with autoclaved water. Subsequently 2 µl each of a dilution series of the peptide (1 mg/ml; 0.1 mg/ml; 0.01 mg/ml; 1 µg/ml; 0.1 µg/ml and without peptide) was added separately on the paper and then the paper was blocked in TBS-T (0.1 M Tris-HCl; 1.5 M NaCl; 0.05% Tween 20; pH 7.4) with 5% skimmed milk powder for 45 min. The blocking buffer was exchanged with the primary antibody in TBS-T and 5% milk powder (1:1000 dilution) and incubated for 1 h, rinsed 3 x 5min with TBS-T before being incubated with the secondary antibody (Anti-guinea Pig IgG-peroxidase antibody produced in rabbit; Sigma Aldrich; diluted 1:50.000) diluted in TBS-T with 5% milk powder for 1 h, washed 2x 5min in TBS-T, followed by 1x 5 min in TBS (0.1 M Tris-HCl; 1.5 M NaCl; pH 7.4). Detection was done with the Clarity Western ECL substrate (Biorad) according to the manufacturer’s instructions. The results clearly showed that the antibody is binding the peptide in question at the 3 highest concentrations.

**Preadsorption negative control**

As a negative control the antibody and the tissue were preadsorbed with the peptide VKAVADHEKEMHNMAKRL before staining by processing the specimen as described in Methods, but after blocking the specimen and before adding the primary antibody, specimen were transferred to blocking solution containing 0,25 mg/ml of peptide and left overnight at 4 °C. Additionally, the r-opsin antibody was treated with the same concentration peptide and left over night at 4 °C before being added to the specimens. Thereafter the specimens were treated as described which lead to no positive signal in the eye region (Figure S6).

**In situ hybridization**

Animals were fixed for 2.5 h in 4 % PFA in phosphate buffer PTW (1X PBS with 0.1 % Tween 20; pH 7.4) at room temperature, rinsed three times in Methanol, followed by three long washes in Methanol (3x 45 min) and stored at -20 °C in Methanol. Specimens were rehydrated in a graded series of 75 %, 50 %, 25 % Methanol in PTW (each step 1-5 min) and washed in PTW (5x5 min). Depending on the developmental stage, animals were digested for 15-45 s with Proteinase K (5 ng in 1 ml PTW), then rinsed twice in 2 mg glycine/ 1 ml PTW, washed for 5 min in acidified 1 % Triethanolamin in PTW (0.5 μl glacial acid/ ml TEA), 2 more minutes after adding another 0.5 μl glacial acid/ ml TEA and then washed again in PTW (2x 5 min). Afterwards specimens were postfixed in 4 % PFA in PTW for 45 min and washed in PTW (5x 5 min). Still remaining in PTW, samples were heated to 80 °C to inactivate Alkaline Phosphatase. Prehybridization in hybridization buffer (50 % Formamide; 5x SSC (0.75 M NaCl, 0.075 M Na citrate); 50 μg/ ml Heparin; 100 μg/ ml SS DNA; 0.1 % Tween20; 1 % SDS; 5 % Dextran sulfate) was done at 65 °C for at least 4 h and hybridization with antisense DIG and/or FITC labelled RNA probes at the same temperature for 72 h. Thereafter animals were transferred via a graded series from hybridization buffer to 2x SSCT (0.1 % Tween 20 in 2x SSC) (75 %, 50 %, 25 %, 2x SSCT, twice 0.05x SSCT; 30 min each step) at 65 °C and then back from SSCT to PTW (75 %, 50 %, 25 %, 5 times PTW – 5 min each step) on room temperature. Samples were blocked for 1h in Roche Blocking solution in maleic acid buffer and subsequently incubated in anti- DIG-AP Fab fragments (Roche) in the blocking solution (1:5000) overnight at 4 °C. Thereafter specimens were washed 6 times within 1 h in PTW, rinsed in AP buffer (0.1 M TrisCl, 0.1 M NaCl, 0.05 M MgCl, 0.1 % Tween 20; pH 8.2) and then transferred to the Fast Blue staining solution (Fast Blue Sigma Alldrich 250 μg/ml; 3-hydroxy-2-naphtoic acid 250 μg/ml in AP buffer). In case of double labellings, specimens were washed in PTW (4x 5min), incubated for 10 min in 0.1 M glycine/ HCl (pH 2.2) on room temperature, washed again in PTW (3x 5min) and blocked once more for 1h in Roche Blocking solution in maleic acid buffer, before being incubated in anti-FITC-AP-Fab fragments (Roche) in the blocking solution (1:5000) overnight at 4 °C. Subsequently, specimens were washed 6 times within an hour in PTW and equilibrated in 0.1 M Tris-Cl (pH 8.2). Detection of anti-FITC AP fragments was performed using Fast Red tablets (Roche) dissolved in 0.1 M Tris-HCl (pH 8.2). After successful staining, specimens were washed in PTW (2x5 min) and stored in the embedding medium (90 % glycerol/ 10% 10x PBS/ 0.25 % DABCO) until usage.

**Electron microscopy**

**Sample preparation.** Larvae were fixed in 2.5 % glutaraldehyde/ 0.1 M sodium cacodylate/ 0.24 M NaCl (pH7.4) for 1 h, washed in EM buffer (0.1 M sodium cacodylate/ 0.24 M NaCl; pH 7.4) and postfixed in 1 % Osmium tetroxide/ 0.1 M sodium cacodylate/ 0.24 M NaCl (pH 7.4) for 30 min. Specimens were en bloc stained in 2 % Osmium tetroxide/ 1 % potassium ferricyanide/ 0.1 M sodium cacodylate (pH 7.4), washed in water and subsequently stained in 2 % aqueous uranyl acetate for 30 min before being dehydrated in a graded ethanol series (50 %, 70 %, 80 %, 90 %, 96 % and 2x 100 %). Ethanol was exchanged with propylene oxide before the animals were embedded in Epon/ Araldite resin and polymerized at 60 °C for 48 h.

**Imaging.** 12 grids were loaded into a single specimen holder and imaged with a Zeiss SUPRA 55VP field-emission scanning electron microscope equipped with a retractable multi-mode transmitted electron detector called (STEM) and the integrated module ATLAS. ATALS enabled automated scan generation, stage translation and serial acquisition of images. Mosaics were set up in ATLAS by determining the desired dimensions of the image and the percentage overlap between image tiles, which was about 6.73 %. The eye region of *L.asellus* was imaged at 28 kV accelerating voltage, 30 μm aperture, 2.1 mm working distance and a dwell time of 1.0 μs per pixel. The field of view was ca. 119 μm and consisted of 4 tiles per single frame with an individual tile resolution at 14834 x 14834 pixels and a pixel size of 8 nm. Upon image acquisition the inverted mode (Q1 = OFF; Q2 and Q3 = INVERTED) was applied in order to obtain TEM like images. In addition, the autofocus function was enabled for every tile while autostigmation for every four tiles. The auto-brightness/ contrast was deactivated while the STEM gain was at medium mode and high current was constantly activated.
