# Supplementary figures and images for: Posterior eyespots in larval chitons have a molecular identity similar to anterior cerebral eyes in other bilaterians
Source: EvoDevo. 2015 Dec 22;6:40. doi: 10.1186/s13227-015-0036-0 (PMC4689004; doi:10.1186/s13227-015-0036-0)

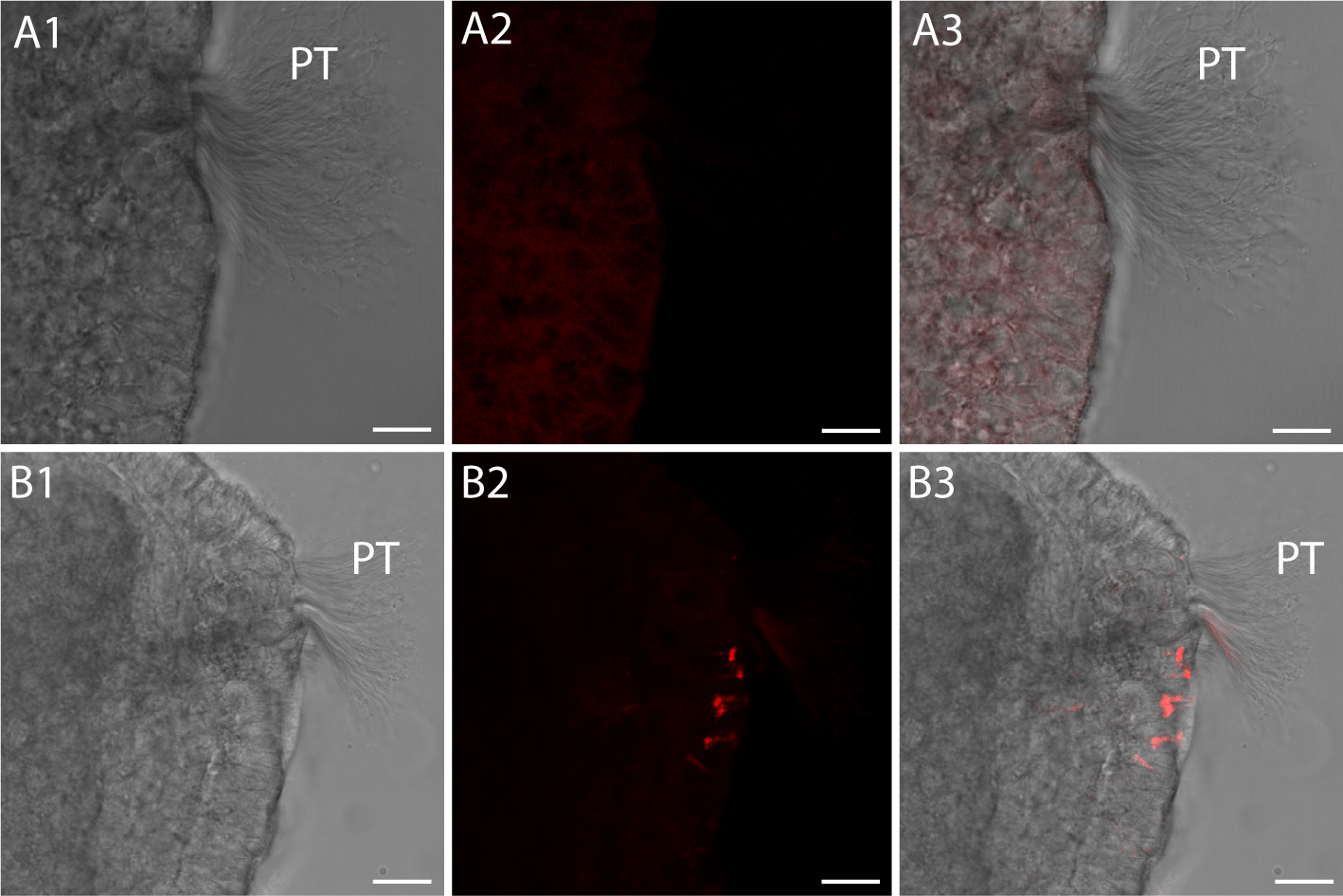

Supplement: Supplementary file 2 — 10.1186/s13227-015-0036-0 R-opsin antibody preadsorbtion test. (A1-A3) The negative control of the specifically designed r-opsin antibody shows no specific signal in the eye region under prototroch (PT) after the specimen and antibody were preadsorbed with the antigenic peptide. (B1-3) The positive control of the antibody shows a clear signal in the eye region (Scalebars: 20 µm in A; 15 µm in B). [file 13227_2015_36_MOESM2_ESM.tif]

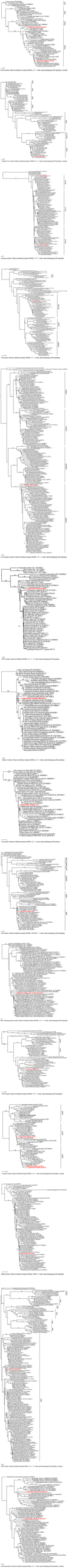

Supplement: Supplementary file 3 — 10.1186/s13227-015-0036-0 Phylogenetic analyses of studied genes with uncertain orthology after reciprocal blast. [file 13227_2015_36_MOESM3_ESM.pdf]

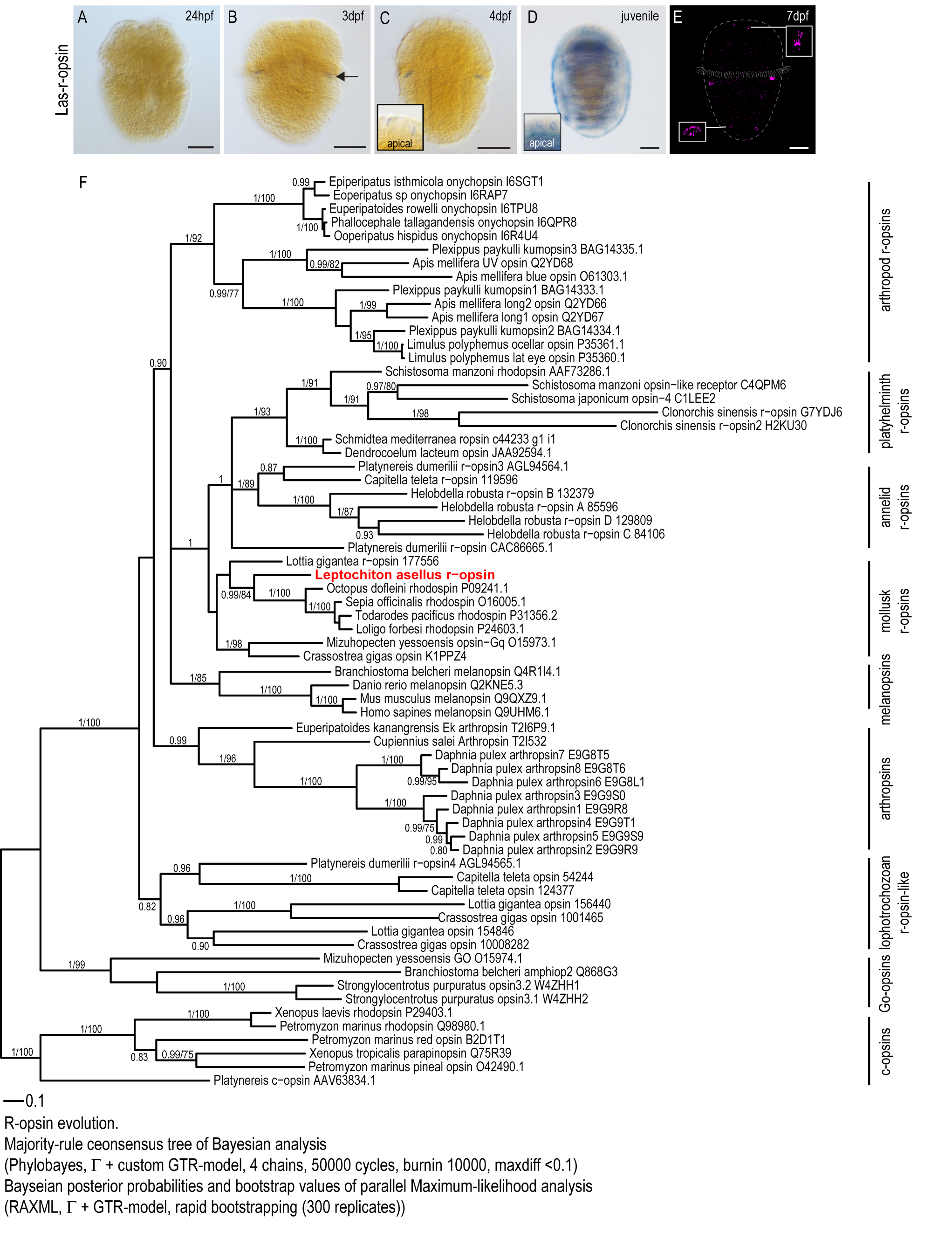

Supplement: Supplementary file 4 — 10.1186/s13227-015-0036-0 Expression of Las-r-opsin in different developmental stages and r-opsin evolution. (A) No expression was found in larvae between 24-48 hpf and first expression was detected in larvae of 3 dpf (B). (C) Clear expression of Las-r-opsin in larvae of 4 dpf and in juvenile animals (D), which also show a background staining in the developing shell. (E) Expression in posterior photoreceptor cells can first be detected in larvae of 7 dpf and only by confocal imaging of fluorescent signal due to low signal strength. (F) Uncollapsed tree of r-opsin analysis shown in Fig. 1. (Scalebars: 100 μm in A-E). [file 13227_2015_36_MOESM4_ESM.tif]

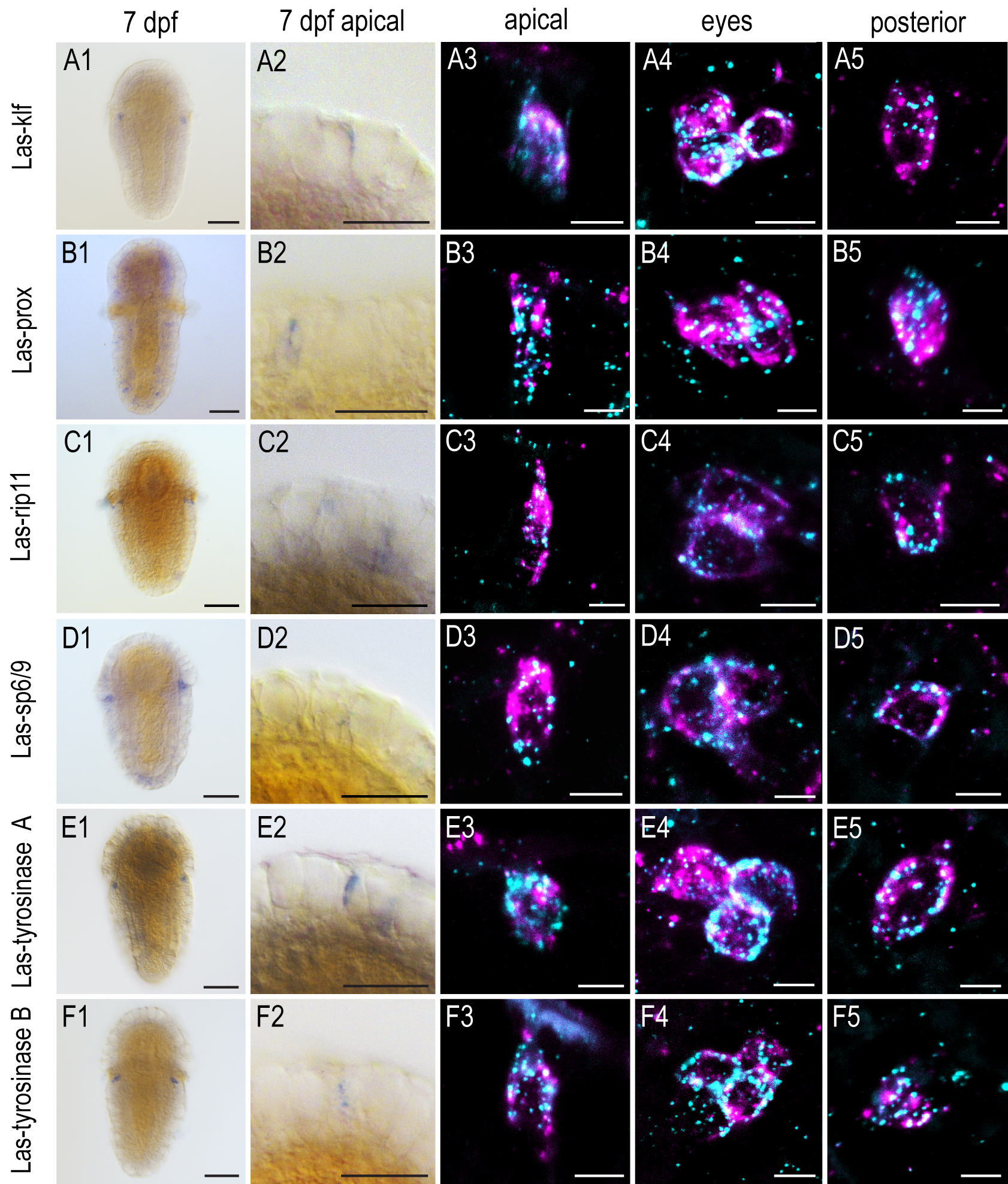

Supplement: Supplementary file 5 — 10.1186/s13227-015-0036-0 Expression of genes involved in PRC development, opsin transport and pigment cell markers. Column 1-2: single labelling of gene X. Column 3-5: double labelling of gene X (cyan) and Las-r-opsin (magenta) in the anterior, posttrochal eye and posterior region. (A1-5) Expression of Las-klf in all PRCs (A1). (B1-5) Expression of Las-prox in all PRCs, as well as in the longitudinal nerve cords (B1). (C1-5) Expression of Rab 11 interacting protein (Las-rip11) in all PRCs. (D1, 3-5) Expression of Las-sp6/9 in all PRCs. (E + F 1-5) Expression of Las-tyrosinase A + B in all PRCs. (Scalebars: 100 μm in column 1; 50 μm in column 2; 5 μm in columns 3-5). [file 13227_2015_36_MOESM5_ESM.tif]

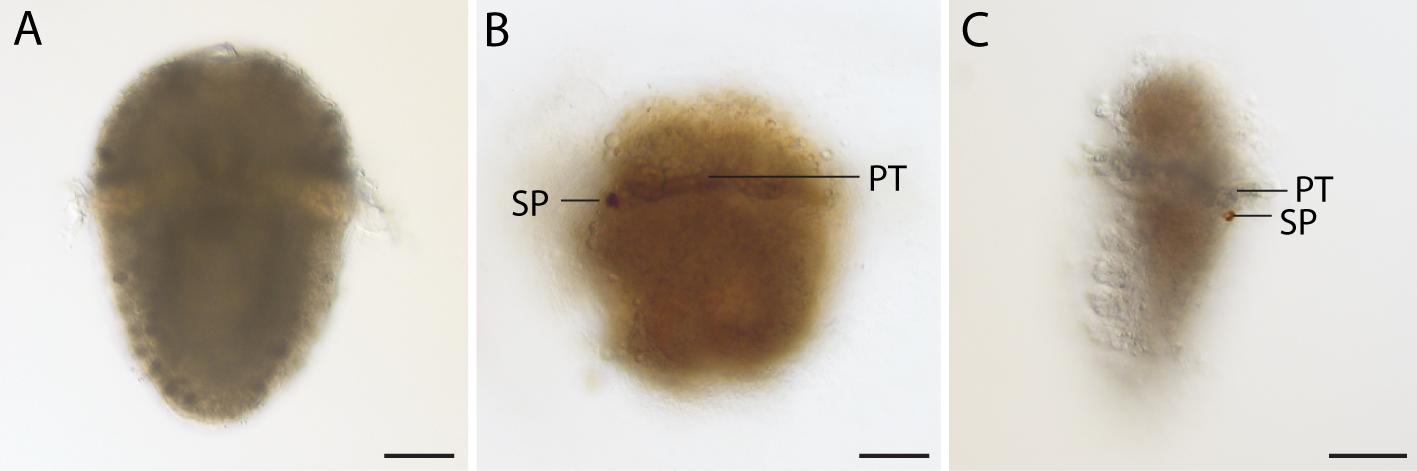

Supplement: Supplementary file 6 — 10.1186/s13227-015-0036-0 Shielding pigment test. (A) After treating the specimen with acidified Methanol the eye pigment spot completely fades. (B) After transferring the animal to 0.1 M NaOH the animal starts disintegrating but the shielding pigment (SP) does not fade and is still clearly visible under the prototroch (PT). The image was taken immediately before the entire animal disintegrated (PT). (C) After transferring the animal to a solution of 10 % Hydrogenperoxide the specimen starts to clear up and disintegrate whereas the shielding pigment remains. The image was taken immediately before the entire animal disintegrated (Scalebares 100 µm). [file 13227_2015_36_MOESM6_ESM.tif]

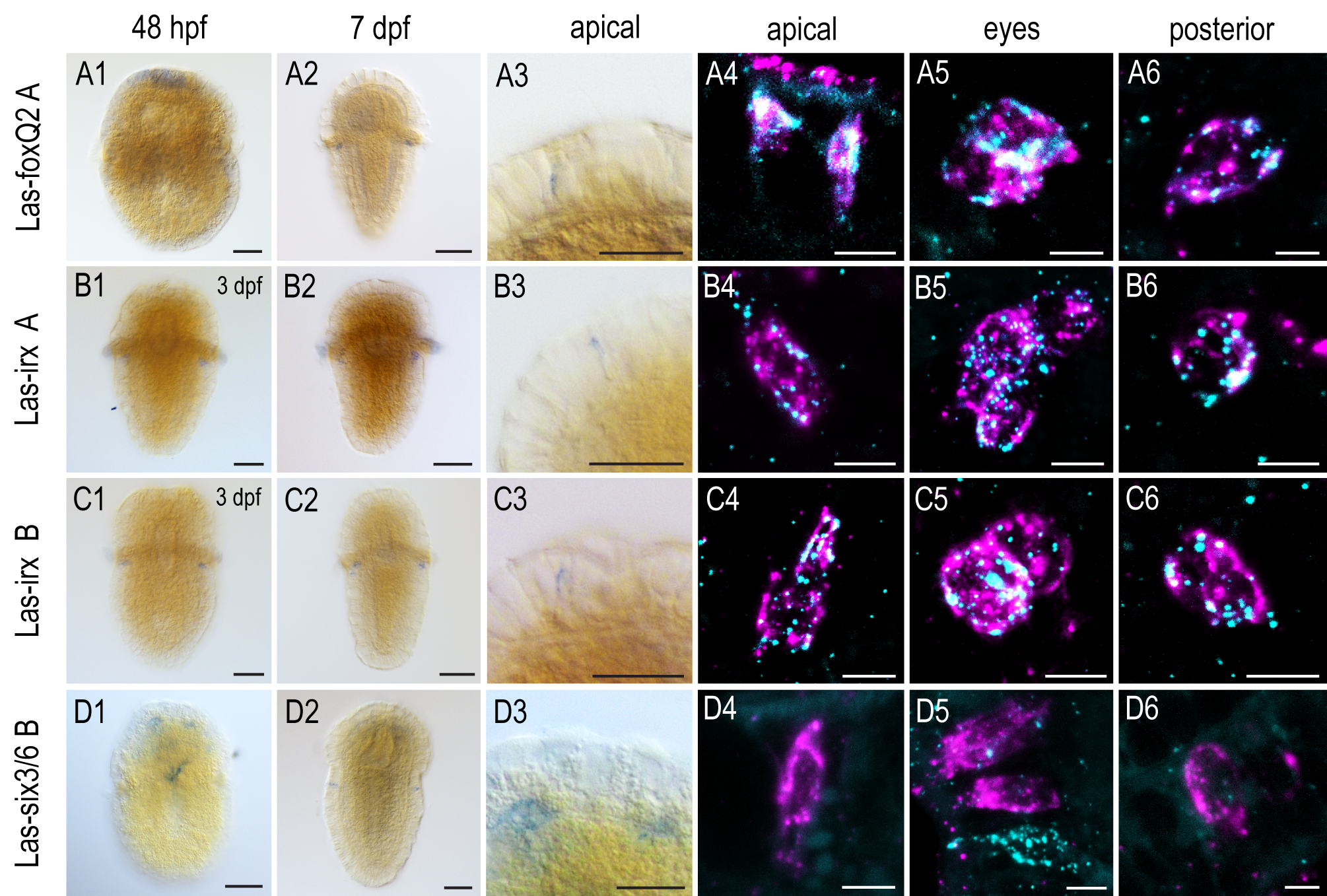

Supplement: Supplementary file 7 — 10.1186/s13227-015-0036-0 Expression of apical markers during the development of L.asellus. Column 1-3: single labelling of gene X. Column 4-6: double labelling of gene X (cyan) and Las-r-opsin (magenta) in the anterior, posttrochal eye and posterior region. (A1-6) Expression of Las-foxq2 A is limited to the apical area only in young larvae (A1) and can be found in all PRCs in older developmental stages. (B + C 1-6) Expression of Las-irx A and B in all PRCs. (E1-6) Las-six3/6 B is expressed in the apical area of young larvae (E1) and a clear expression can be found separate from the apical area underneath the eye region in older larvae (E2, E 5). No expression was found in the apical (E4) or posterior (E6) r-opsin + cells. (Scalebars: 100 μm in columns 1,2; 50 μm in column 3; 5 μm in columns 4-6). [file 13227_2015_36_MOESM7_ESM.tif]
